# Immunogenicity and protective efficacy of inactivated SARS-CoV-2 vaccine candidate, BBV152 in rhesus macaques

**DOI:** 10.1038/s41467-021-21639-w

**Published:** 2021-03-02

**Authors:** Pragya D. Yadav, Raches Ella, Sanjay Kumar, Dilip R. Patil, Sreelekshmy Mohandas, Anita M. Shete, Krishna M. Vadrevu, Gaurav Bhati, Gajanan Sapkal, Himanshu Kaushal, Savita Patil, Rajlaxmi Jain, Gururaj Deshpande, Nivedita Gupta, Kshitij Agarwal, Mangesh Gokhale, Basavaraj Mathapati, Siddhanath Metkari, Chandrashekhar Mote, Dimpal Nyayanit, Deepak Y. Patil, B. S. Sai Prasad, Annasaheb Suryawanshi, Manoj Kadam, Abhimanyu Kumar, Sachin Daigude, Sanjay Gopale, Triparna Majumdar, Deepak Mali, Prasad Sarkale, Shreekant Baradkar, Pranita Gawande, Yash Joshi, Sidharam Fulari, Hitesh Dighe, Sharda Sharma, Rashmi Gunjikar, Abhinendra Kumar, Kaumudi Kalele, Vellimedu K. Srinivas, Raman R. Gangakhedkar, Krishna M. Ella, Priya Abraham, Samiran Panda, Balram Bhargava

**Affiliations:** 1 Indian Council of Medical Research-National Institute of Virology, Pune, 411021 Maharashtra India; 2 Bharat Biotech International Limited, Genome Valley, Hyderabad, 500 078 Telangana India; 3 Department of Neurosurgery, Command Hospital (Southern Command), Armed Forces Medical College (AFMC), Pune, 411040 Maharashtra India; 4 Army Institute of Cardio-Thoracic Sciences, Pune, 411040 Maharashtra India; 5 Indian Council of Medical Research, V. Ramalingaswami Bhawan, New Delhi, 110029 India; 6 ICMR-National Institute for Research in Reproductive Health, Mumbai, 400012 Maharashtra India; 7 Department of Veterinary Pathology, Krantisinh Nana Patil College of Veterinary Science, Shirwal, 412801 Maharashtra India

**Keywords:** Inactivated vaccines, SARS-CoV-2, Viral infection, Preclinical research

## Abstract

The COVID-19 pandemic is a global health crisis that poses a great challenge to the public health system of affected countries. Safe and effective vaccines are needed to overcome this crisis. Here, we develop and assess the protective efficacy and immunogenicity of an inactivated SARS-CoV-2 vaccine in rhesus macaques. Twenty macaques were divided into four groups of five animals each. One group was administered a placebo, while three groups were immunized with three different vaccine candidates of BBV152 at 0 and 14 days. All the macaques were challenged with SARS-CoV-2 fourteen days after the second dose. The protective response was observed with increasing SARS-CoV-2 specific IgG and neutralizing antibody titers from 3^rd^-week post-immunization. Viral clearance was observed from bronchoalveolar lavage fluid, nasal swab, throat swab and lung tissues at 7 days post-infection in the vaccinated groups. No evidence of pneumonia was observed by histopathological examination in vaccinated groups, unlike the placebo group which exhibited interstitial pneumonia and localization of viral antigen in the alveolar epithelium and macrophages by immunohistochemistry. This vaccine candidate BBV152 has completed Phase I/II (NCT04471519) clinical trials in India and is presently in phase III, data of this study substantiates the immunogenicity and protective efficacy of the vaccine candidates.

## Abstract (web summary)

SARS-CoV-2 vaccines are needed to fight the pandemic. Here, authors show protective efficacy and immunogenicity of the inactivated SARS-CoV-2 vaccine BBV152 in rhesus macaques. This vaccine is currently in clinical development.

## Introduction

The pandemic of coronavirus disease 2019 (COVID-19) has caused an unprecedented public health burden in several countries across the globe^1^. The spread of severe acute respiratory syndrome coronavirus-2 (SARS-CoV-2) is humongous affecting more than 75 million people till December 20, 2020^2^. With all the public health interventions in place including behavioural modifications such as the use of mask, hand sanitisation and social distancing, pharmaceutical interventions such as antiviral drugs or vaccines seem to be the only means of stopping this raging pandemic. With the release of the first genome sequence of SARS-CoV-2 from China on 11 January 2020, the race against the virus and time had begun for the development of an effective COVID-19 vaccine. Multiple vaccine development platforms from traditional to next-generation approaches are being used by different research groups worldwide. Purified inactivated viruses have been traditionally used in vaccine development. The inactivated vaccines have been proven to be safe and effective in the prevention of diseases like rabies, polio, hepatitis A and influenza^3^. Production of inactivated vaccine is relatively easy and the speed, with which it can be made, makes it a promising strategy for vaccine development for COVID-19^4^. Earlier experience of inactivated vaccine manufacturing and the availability of a BSL-3 facility equipped with a well-characterised Vero cell manufacturing platform aided us in the rapid development of an inactivated vaccine candidate. Moreover, two similar inactivated SARS-CoV-2 candidates based on alum adjuvant, i.e., PiCoVacc and BBIPCorV, have shown encouraging results in non-human primate (NHP) models and have progressed to the human clinical trials^5,6^.

The safety and the immunogenicity of inactivated vaccine candidate BBV152 has been established in mice, rat and rabbit models^7^.The vaccine candidate has been found to induce an effective humoral immune response and protects Syrian hamsters from SARS-CoV-2 pneumonia^8^. Here, we report the assessment of immunogenicity and protective efficacy of three formulations of a purified whole-virion-inactivated SARS-CoV-2 vaccine candidate BBV152 in the rhesus macaques.

## Results

### Anti-SARS-CoV-2 IgG and neutralising antibody response

**Fig. 1:**
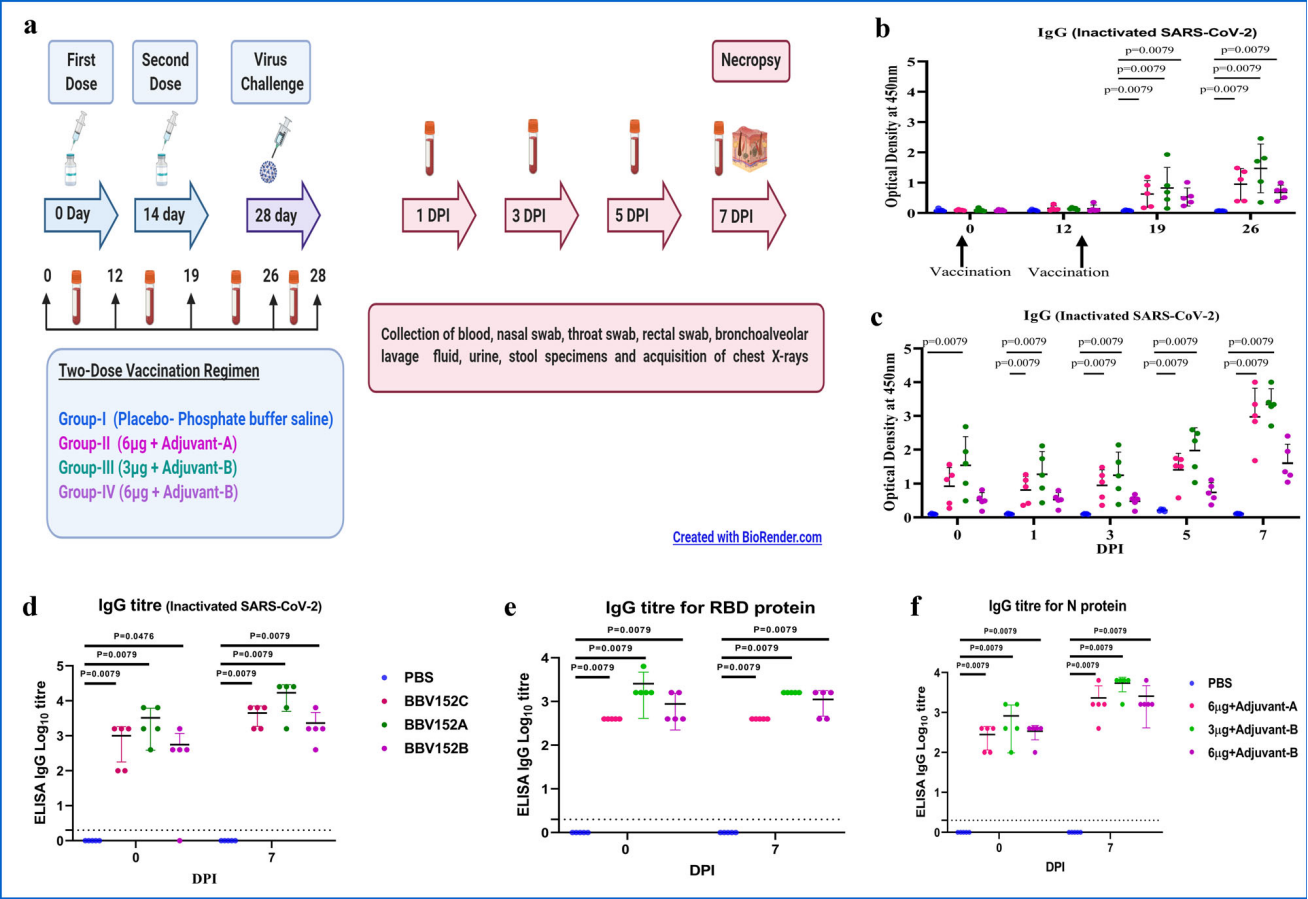
Experimental summary and IgG response in vaccinated animals. **a** Diagrammatic representation of experiment summary. **b** Anti-SARS-CoV-2 IgG response in animals from 1st to 4th week of immunisation with whole virus-inactivated protein ELISA. **c** Anti-SARS-CoV-2 IgG response at 0, 1, 3, 5 and 7 DPI. **d** Anti-SARS-CoV-2 IgG titres in animals at 0 and 7 DPI. **e** IgG titres for SARS-CoV-2 RBD protein in animals at 0 and 7 DPI. **f** IgG titres for SARS-CoV-2 nucleoprotein in animals at 0 and 7 DPI. The statistical significance was assessed using the Kruskal–Wallis test followed by the two-tailed Mann–Whitney test between two groups; *P* values of < 0.05 were considered to be statistically significant. The dotted line on the figures indicates the limit of detection of the assay. Data are presented as mean values +/− standard deviation (SD). Statistical comparison was done by comparing the vaccinated group with the placebo group as a control. Group I = blue, group II = pink, group III = green and group IV = purple, number of animals = 5 animals in each group. Source data are provided as a Source Data file.

Adverse events were not seen in animals immunised with a two-dose vaccination regimen. We evaluated anti-SARS-CoV-2 immunoglobulin-G (IgG) antibody and neutralising antibody (NAb) titres from the serum samples during the immunisation phase on 0, 12, 19, 26 and 28 days and after SARS-CoV-2 infection on 0, 1, 3, 5 and 7 days post infection (DPI) (Fig. 1a). Using inactivated SARS-CoV-2 IgG antibody detection ELISA, IgG levels were detectable from 3rd week post immunisation and were found increasing till 35th day (7 DPI) (Fig. 1b, c). Group III animals showed the highest IgG titre (1:25,600) compared to groups II and IV (1:1600–1:6400) (Fig. 1d).

Anti-SARS-CoV-2 IgG specific to RBD protein showed a high level of antibody response in the vaccinated group at 28th day post immunisation and 7 DPI (Fig. 1e). Group III showed the highest titre of RBD IgG antibody (1:1600) compared to groups II (1:400) and IV (1:400–1:1600). Similarly, N protein-based ELISA indicated higher IgG titre (1:6400) in Group III as compared to groups II (1:400–1:6400) and IV (1:1600–1:6400) (Fig. 1f).

**Fig. 2:**
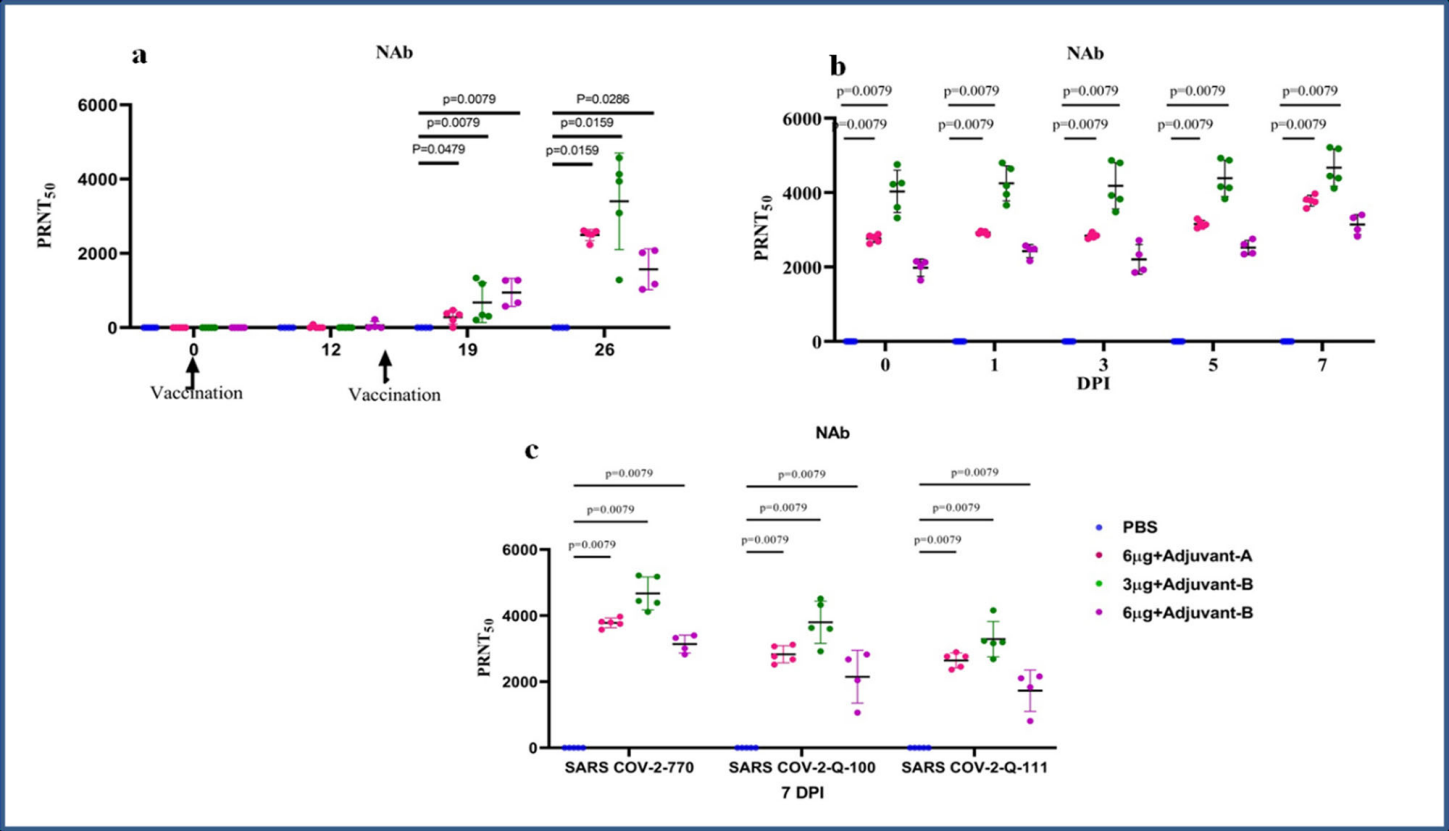
NAb response in vaccinated animals. **a** NAb titres in animals from 1st to 4th week of immunisation. **b** NAb titres in animals at 0, 1, 3, 5 and 7 DPI. **c** NAb titres in animal samples of 7 DPI with homologous strain 770 and heterologous strain Q-100 and Q-111 of SARS-CoV-2. The statistical significance was assessed using the Kruskal–Wallis test followed by the two-tailed Mann–Whitney test between two groups; *P* values of < 0.05 were considered to be statistically significant. Data are presented as mean values +/− standard deviation (SD). Statistical comparison was done by comparing the vaccinated group with the placebo group as a control. Group I = blue, group II = pink, group III = green and group IV = purple, number of animals = 5 animals in each group. Source data are provided as a Source Data file.

The highest NAb titres of 1:209–1:5217 were detected in group III after the SARS-CoV-2 challenge. The NAb titres for groups II and IV were (1:87.4–1:3974) and (1:29.5–-72 h:3403), respectively (Fig. 2a, b). These NAb titres correlated with the IgG antibody titres. NAb and IgG response was not detectable in the placebo group. Both homologous and heterologous SARS-CoV-2 strains (Q-100 and Q-111) were found to be neutralised by the macaque serum samples collected on 7 DPI (Fig. 2c).

### Viral load in the nasal swab, throat swab and bronchoalveolar lavage fluid

**Fig. 3:**
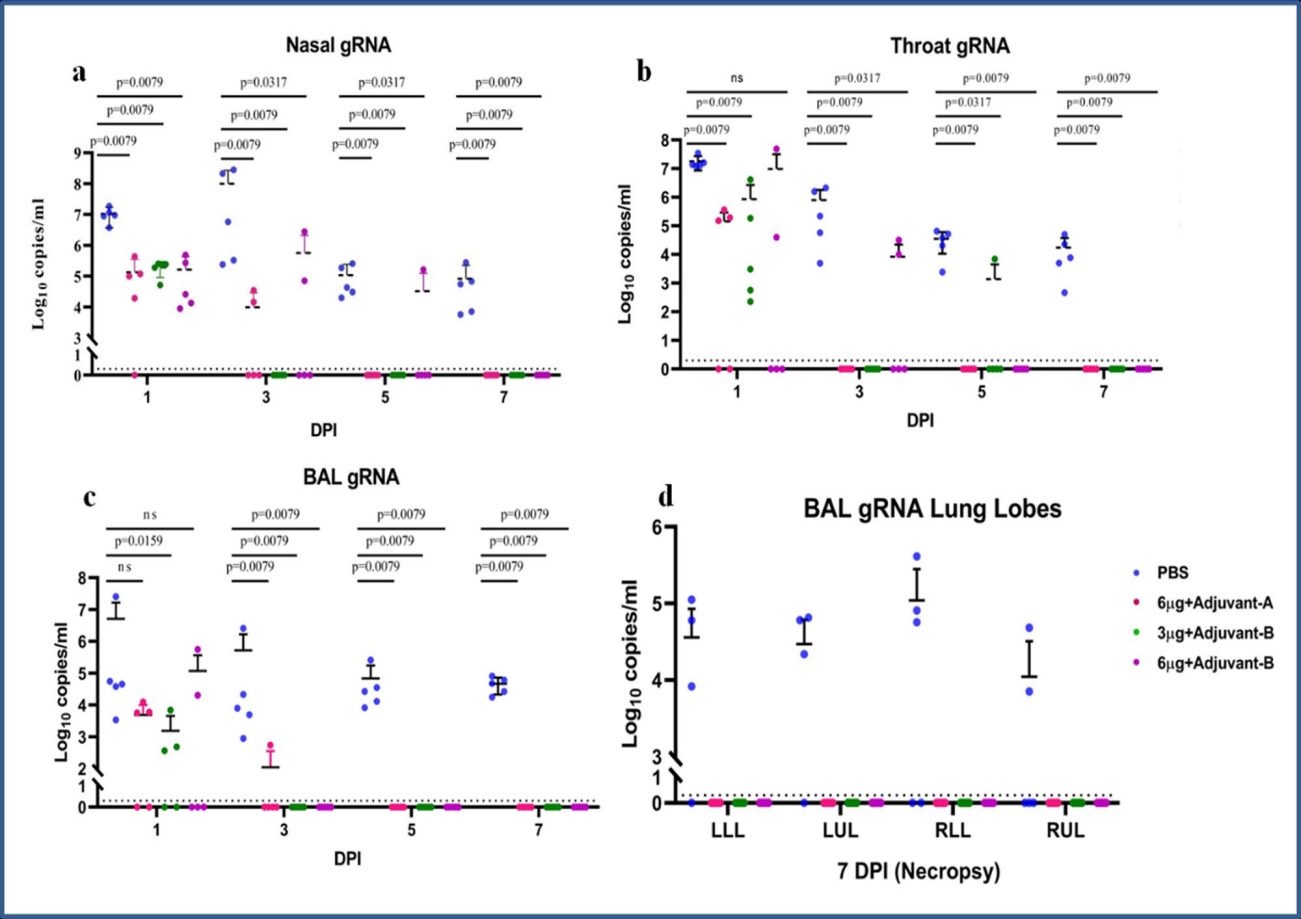
Genomic viral RNA detection in respiratory tract specimens. Genomic viral RNA load in (**a**) nasal swab, (**b**) throat swab and (**c**) BAL at 1, 3, 5 and 7 DPI. **d** Genomic viral RNA load in different lobes of lungs at 7 DPI. The statistical significance was assessed using the Kruskal–Wallis test followed by the two-tailed Mann–Whitney test between the two groups; *P* values < 0.05 were considered to be statistically significant. The dotted lines indicate the limit of detection of the assay. Data are presented as mean values +/− standard deviation (SD). Statistical comparison was done by comparing the vaccinated group with the placebo group as a control. Group I = blue, group II = pink, group III = green and group IV = purple, number of animals = 5 animals in each group. Source data are provided as a Source Data file.

Genomic RNA (gRNA) was detected from nasal swab (NS) specimens of all animals in the placebo group from 1 to 7 DPI. Viral clearance was observed in NS specimens of all the animals from vaccinated groups on 7 DPI (Fig. 3a).

Throat swab (TS) specimens of the placebo group were tested positive for gRNA at 1, 3, 5 and 7 DPI. Vaccinated groups had a detectable level of gRNA from 1 to 5 DPI with complete viral clearance on 7 DPI (Fig. 3b). Bronchoalveolar lavage (BAL) fluid specimens of the animals from the placebo group were positive for gRNA from 1 to 7 DPI. In the vaccinated groups, gRNA was detected in BAL specimens until 3 DPI (Fig. 3c). Except for the placebo group, none of the vaccinated groups showed the presence of gRNA in lung lobes in necropsy tissue (Fig. 3d). The comparisons of viral copy numbers of the NS, TS and the BAL fluid samples of the vaccinated as compared to the placebo group were found to be statistically significant using the two-tailed Mann–Whitney test.

**Fig. 4:**
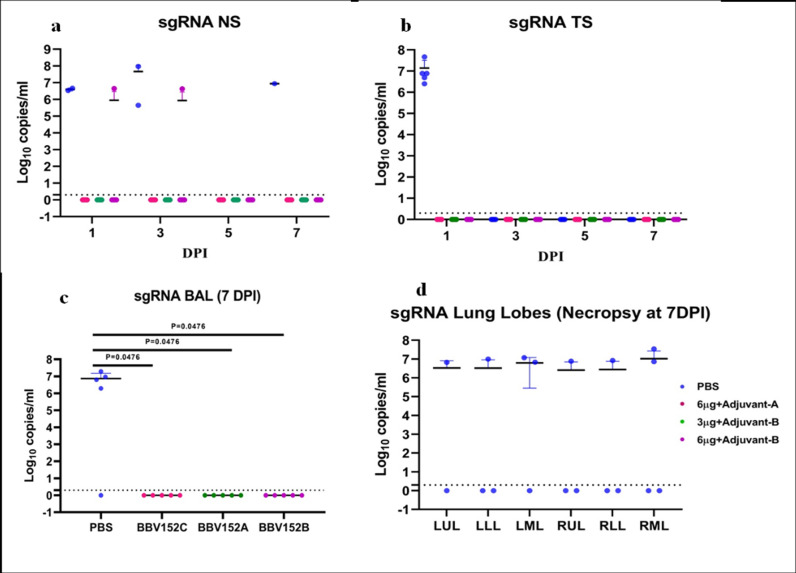
Subgenomic viral RNA detection in the respiratory tract specimens. sgRNA load in (**a**) BAL at 7 DPI, (**b**) sgRNA in different lobes of lungs at 7 DPI, (**c**) throat swab at 1 DPI and (**d**) sgRNA load in NS at 1, 3, 5 and 7 DPI. The statistical significance was assessed using the Kruskal–Wallis test followed by the two-tailed Mann–Whitney test between the two groups; *P* values < 0.05 were considered to be statistically significant. The dotted lines indicate the limit of detection of the assay. Data are presented as mean values +/− standard deviation (SD). Statistical comparison was done by comparing the vaccinated group with the placebo group as a control. Group I = blue, group II = pink, group III = green and group IV = purple, number of animals = 5 animals in each group. Source data are provided as a Source Data file.

Subgenomic RNA was detected in the BAL fluid of 4/5 animals of the placebo animals, whereas it was negative for all the vaccinated animals at 7 DPI (Fig. 4a). In the necropsy specimens of lung tissue at 7 DPI, sgRNA was detected in multiple lobes of 4/5 placebo group of animals (three lobes in 1/5, two lobes in 2/5 and one lobe in 1/5 animals), whereas no sgRNA could be detected in the lung tissue of vaccinated animals (Fig. 4b). In the TS of animals of the placebo group, sgRNA was detected in all the animals at 1 DPI, whereas all the vaccinated animals were negative for sgRNA (Fig. 4c). In placebo-group animals, sgRNA was detected in NS of 2/5 animals at 1 DPI, 2/5 at 3 DPI and 1/5 at 7 DPI. In the vaccinated group, there were significantly lower numbers of animals positive for sgRNA in NS even at 1 DPI (Fig. 4d).

The presence of sgRNA in the BAL fluid and lung tissue (at necropsy) of animals from placebo group I at 7 DPI and absence in the vaccinated animals demonstrates the protective efficacy of the vaccine candidates.

### Viral load in the respiratory tract, lungs and extrapulmonary organs

**Fig. 5:**
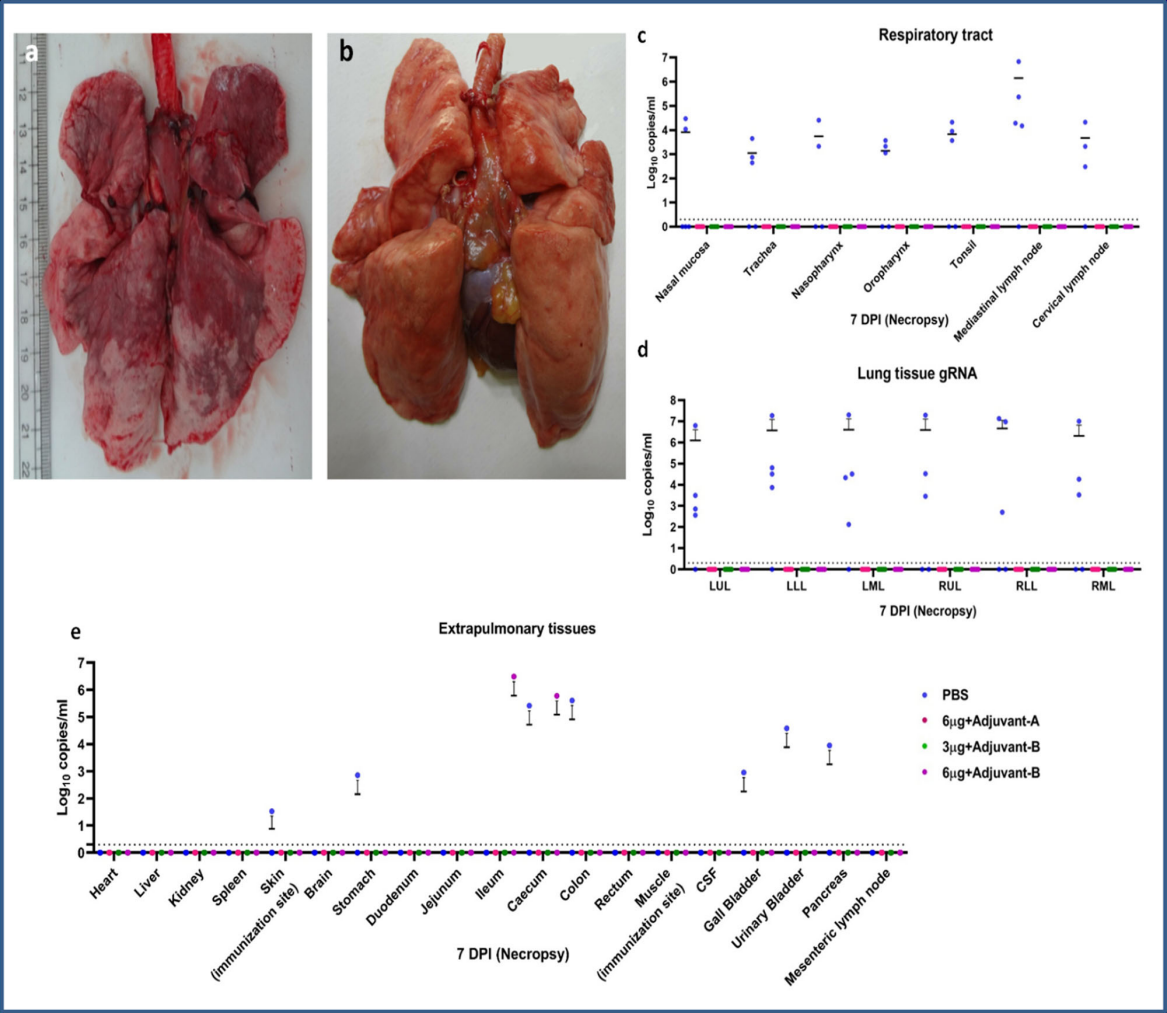
Gross pathology of lungs and viral RNA load in different tissues. **a** Lungs showing extensive involvement of the right upper lobe (RUL), right lower lobe (RLL), left upper lobe (LUL) and left lower lobe (LLL) (group I) and (**b**) normal lung (group III) (**c**) genomic viral RNA of respiratory tract tissues (**d**), lungs tissue and (**e**) extrapulmonary organs at 7 DPI. The dotted lines indicate the limit of detection of the assay. Group I = blue, group II = pink, group III = green and group IV = purple, number of animals = 5 animals in each group. Source data are provided as a Source Data file.

On 7 DPI, animals from all the groups were sacrificed and swab samples, BAL fluid and various organs were collected. In the placebo group, gRNA was detected in the trachea (3/5), nasopharyngeal mucosa (2/5), oropharyngeal mucosa (3/5) and nasal mucosa (1/5) specimens (Fig. 5c). Four out of five animals of the placebo group had detectable gRNA and sgRNA in multiple lobes of lungs (Figs. 5d and 4b). Lung specimens of all animals from the vaccinated groups were found negative for gRNA and sgRNA. In the placebo group, gRNA was detected in the skin, ileum, colon, gall bladder, stomach, urinary bladder and pancreas. Only one animal from group IV showed the presence of gRNA in ileum and caecum, but it was negative for sgRNA. The heart, liver, kidney, spleen and brain were tested negative for gRNA and sgRNA in all animals (Fig. 5e).

### Clinicoradiological analysis

Weight loss, pyrexia, worsening of SpO_2_ at room air, lethargy, reduced food/water intake and reduced self-grooming were observed in the placebo group, which persisted till 7 DPI, whereas these features resolved in the other groups II and IV (Supplementary Fig. 1 and Supplementary Table 1). Chest radiograph of the three animals in the placebo group showed infiltrates that are suggestive of bronchopneumonia/lobar pneumonia that persisted till 7 DPI. Similarly, chest radiographic abnormalities were detected in two out of five animals in groups II and IV, but resolved by 5 DPI (Supplementary Fig. 2a–d). No clinical or radiographic abnormalities were detected in group III animals.

### Histopathological examination and immunohistochemistry

**Fig. 6:**
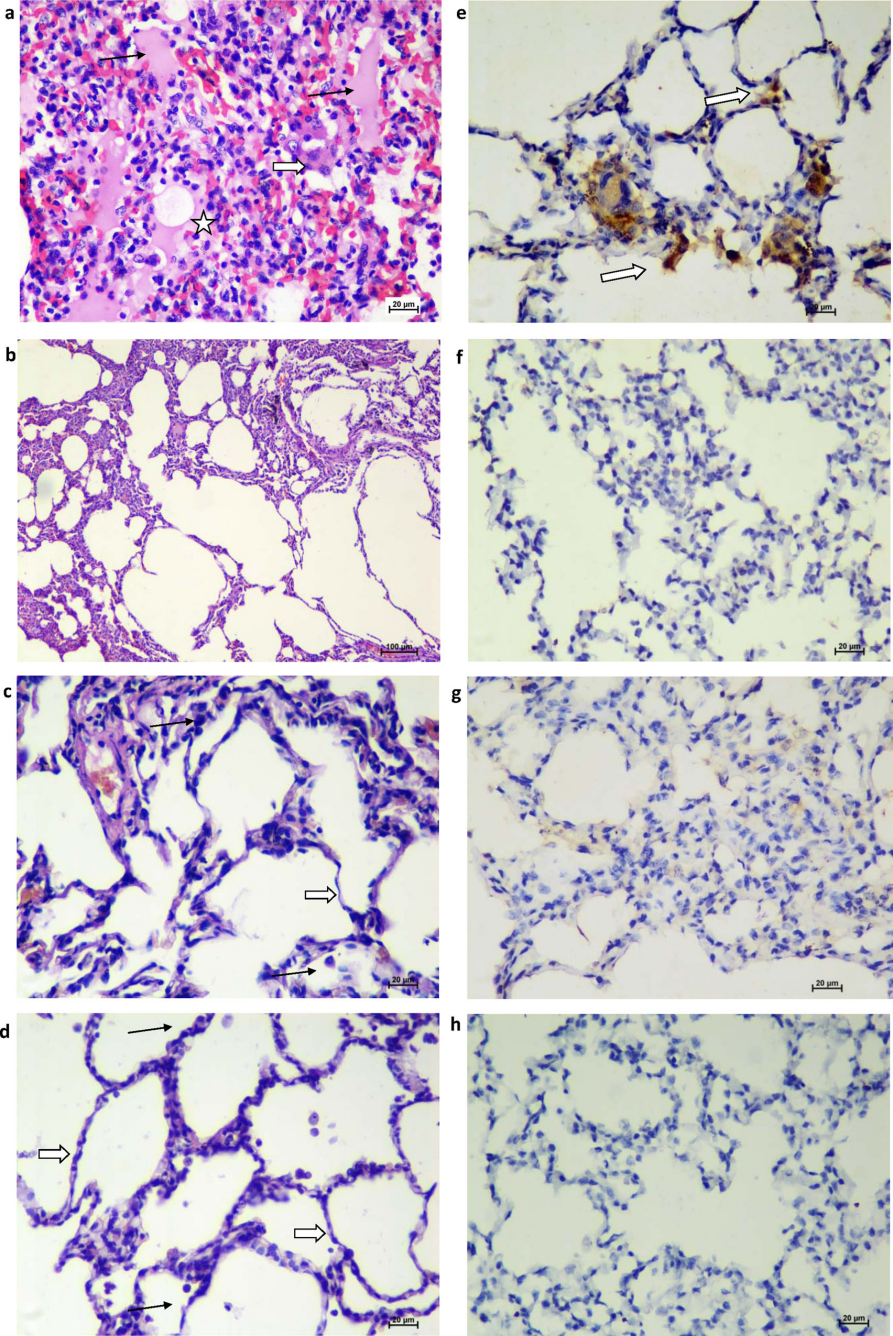
Histopathological and immunohistochemical findings. **a** Lungs showing moderate acute inflammatory changes with haemorrhages and infiltration of inflammatory cells. Alveolar septa showed hyaline membrane formation (asterisks) with inflammatory cells and RBCs. The adjacent alveolar interstitium is thickened by oedema (black arrow) and moderate infiltration of lymphocytes, neutrophils and macrophages (white broad arrow). Haematoxylin and eosin-stained (H&E), ×400 magnification. **b** Lungs showing normal histomorphological features of alveolar septa with type I pneumocyte (white arrow) and occasional alveolar macrophages (black arrow) along the alveolar lumen in group II H&E, ×400 magnification. **c** Lung section depicting normal histomorphological features of alveolar septa with type I pneumocyte (white arrow) and occasional alveolar macrophages (black arrow) along the alveolar lumen in group III H&E, ×400 magnification. **d** Lung section depicting normal histomorphological features of alveolar septa with type I pneumocyte (white arrow) and occasional alveolar macrophages (black arrow) along the alveolar lumen in group IV H&E, ×400 magnifications. **e** Lungs section of placebo group showing the presence of viral antigen by immunohistochemistry (IHC) in type-I pneumocytes (arrow) of alveolar septa and in alveolar macrophages (arrow) (×400 magnification). Lung section of group II (**f**), group III (**g**) and group IV (**h**) showing the absence of viral antigen by IHC. Sixty sections of lung lobe are evaluated for five animals each from groups I, II, III and IV; a representative lesion from each group was selected for the figure.

The gross pathology of the lungs of animals of the placebo group at 7 DPI showed a significantly higher incidence of bronchopneumonic patches and consolidation at necropsy, as compared to the vaccinated groups (Fig. 5a, b). Lung tissues showed moderate-to-severe interstitial pneumonia in the animals of the placebo group (Supplementary Table 2) characterised by thickening of alveolar septa, hyaline membrane formation, accumulation of oedematous fluid and fibrin. Occasionally, certain foci of bronchioles showed necrosis and loss of epithelium with neutrophils and macrophage infiltration (Fig. 6a). Moderate-to-severe disease was present in 3/5 and mild disease in 2/5 animals of the placebo group with the involvement of two to six lung lobes. In group II, 2/5 animals had a single lobe of the lung showing focal mononuclear infiltration (Supplementary Table 3). Lungs of all the other animals of groups II, III and IV appeared normal on histopathology (Fig. 6b–d). Viral antigens were detected in the alveolar epithelium by immunohistochemistry (IHC) in the placebo group suggestive of SARS-CoV-2 infection (Fig. 6e), whereas similar findings were not present in the vaccinated group animals. These findings indicate significant protection of the lungs of the vaccinated group compared to the placebo group (Fig. 6f–h). Groups II, III and IV had significantly lower disease burden compared to the placebo group (Supplementary Tables 2 and 3).

### Virus isolation

NS, TS, BAL fluid, urine, stool and lung tissue specimens from placebo (1–7 DPI) and vaccinated groups were processed for virus isolation. Cytopathic effect (CPE) was observed in TS and NS specimens of placebo group on 1 and 3 DPI. BAL, urine, stool and lung specimens did not show CPE in any group. Two TS and one NS specimen of groups II and IV, respectively, yielded virus isolation on 1 DPI.

### Lymphocyte subset and cytokine/chemokine profile

Lymphocytes were found to be low in the placebo (mean ± SD, 14.51 ± 9.31) compared to the vaccinated macaques (group II, mean ± SD, 31.88 ± 15.85) on 1 DPI. The assessment based on the surface marker of gated lymphocytes from lysed whole blood showed no statistically significant difference between placebo and vaccinated groups in terms of the percentage of cells expressing CD3+ and CD20+ till 7 DPI (extended data, Figs. 3 and Fig. 4). IL-6 was found to be higher in the placebo group (mean ± SD, 22.78 ± 23.21) compared to groups II (mean ± SD, 2.97 ± 4.85) and III (mean ± SD, 2.65 ± 1.98). In addition, the anti-inflammatory cytokine IL-5 was significantly higher in group II (mean ± SD, 3.14 ± 2.41, *P* < 0.05) and group III (mean ± SD, 3.86 ± 3.33, *P* < 0.05) compared to the placebo group on 1 DPI. IL-8 was found significantly higher in group III on 1 DPI, 3 DPI and 5 DPI (mean ± SD, 3472 ± 3017, *P* < 0.05, mean ± SD, 4653.65 ± 3088.34, *P* < 0.05, mean ± SD, 4715.5 ± 2864.15, *P* < 0.01) compared to the placebo group (mean ± SD, 1891.16 ± 878.11, 3 DPI, mean ± SD, 1216.11 ± 757.64, 5 DPI). IL-2, TNF-α and IFN-γ were not detectable in all the animals (extended data, Fig. 5).

## Discussion

A safe and effective vaccine is the need of the hour to overcome the COVID-19 pandemic. In the global race for the development of vaccines, few research groups have reported the preclinical studies of viral vectored vaccines (ChAdOx1 nCoV-19 and Ad-26.COV.2.S)^9,10^, mRNA vaccine (mRNA-1273)^11^, DNA vaccine (INO-4800)^12^ and inactivated vaccines (PiCoVacc and BBIBP-CorV)^5,6^. Phase III clinical trials have been completed for (mRNA-1273)^11^ and BNT162b2 (BioNTech/Pfizer)^13^ vaccine candidates and have been granted Emergency Use Authorisation (EUA) by FDA in USA^14^. The other vaccine candidates are in the advanced stage of phase III clinical trials. Here, we report the two-dose vaccination regimen of inactivated SARS-CoV-2 vaccine candidates, which was found to induce a strong immune response and protection of animals from the infection of SARS-CoV-2.

NAbs and IgG responses were observed from 3^rd^week post immunisation in vaccinated groups. IgG titres were incremental with the highest response in group III. The presence of gRNA in NS was observed in the placebo group till 7 DPI. Vaccinated groups had no detectable gRNA in NS on 7 DPI indicating the ability of vaccine candidates to limit upper respiratory tract viral replication, which is a key factor determining the virus transmission. gRNA was not detected in the BAL fluid from 5 DPI, suggesting that vaccination hindered virus replication and enabled faster clearance from lower airway protecting the animals. gRNA was detected in multiple organs at necropsy in the placebo group, whereas it was found to be cleared in the vaccinated groups.

The presence of sgRNA in the respiratory tissues has been considered as a marker of active replication of the SARS-CoV-2 in the lungs^15^. However, Alexandersen et al.^16^ has suggested that the detection of sgRNA is not direct evidence of active infection; instead, its presence is just detected at lower levels than virion gRNA resulting in detection for a shorter period of time. The detection of sgRNA in the tissues will be influenced by the volume of virus challenge dose and the TCID50 value of the virus used for the challenge. In the NHP preclinical studies for SARS-CoV-2 vaccine candidates published showing significant detection of sgRNA, the volumes of virus used during the challenge study have been varying. In ChAdOx1-nCoV NHP challenge study^9^, virus challenge dose used was 6.5 ml and in mRNA-1273 (Moderna) NHP challenge study^11^ volume of virus used was 4 ml. The protective efficacy of inactivated vaccine candidates BBIPCorV^5^, PiCoVacc^6^ and BNT162b2, an mRNA vaccine candidate^13^, has been demonstrated in these NHP studies even though sgRNA was not evaluated. In our study, the total volume of virus used for challenge was 1.5 ml (intratracheal 1 ml, intranasal 0.5 ml, TCID50 10^6.5^/ml). This may explain the lower rate of detection sgRNA in the post-challenge period. However, even with a lower volume of virus used for the challenge, sgRNA was detected in multiple lobes of 4/5 animal in the placebo group in the necropsy specimen of lungs at 7 DPI, while sgRNA was absent in all the animals of the vaccinated group demonstrating the protective efficacy of the vaccine candidates.

Another factor that needs to be highlighted is the volume of fluid instilled prior to collection of BAL fluid. At 7 DPI, we instilled 10–15 ml of saline to aspirate BAL fluid prior to necropsy and sgRNA was detected in 4/5 animals of the placebo group, while the vaccinated animals had no detection of sgRNA in BAL fluid. In the BAL fluid, aspiration procedure performed at 1, 3 and 5 DPI, we had instilled only 3–5 ml of saline prior to aspiration, which explains the poor detection of the sgRNA in the initial samples. The presence of sgRNA in the BAL fluid at 7 DPI and lung tissue at necropsy of the animals in the placebo group and absence in the vaccinated groups demonstrates the protective efficacy of the vaccine candidates investigated in this study.

Wang et al. and colleagues reported immunogenicity of the two-dose vaccination regimen with effective NAb response in low and middle vaccine doses of inactivated SARS-CoV-2 vaccine and noted virus clearance^5^. Similarly, Gao et al. and colleagues also reported rising titres of NAb with three dose vaccination regimen and protection of animals by virus clearance from the pharynx or lungs at 7 DPI with high vaccine dose^6^.

An elevated level of IL-6 is highly consistent in COVID-19 disease and lymphopenia may be associated with a high level of IL-6^17,18^. Our experimental data showed an elevated level of IL-6 in the placebo group compared to vaccinated groups (II and III) on 1 DPI, suggestive of protection against the SARS-CoV-2 infection. The transient lymphopenia in the placebo group could be suggestive of suboptimal cellular immunity in the placebo group or may be due to the recruitment of lymphocytes to the inflamed respiratory tract^19,20^. Furthermore, lymphopenia is specifically CD8+ T cells biased^19,21^, which possibly explains the elevated proportion of CD4+ T cells in the placebo group^19^. IL-5, a Th_2_ cytokine, is associated with eosinophilia and evidence of eosinophils for antiviral activity is documented^22^. Our study demonstrated a transient increase in serum IL-5 level on 1 DPI in the vaccinated group II, which subsided by 3 DPI. In addition, the haematological data indicated the percentage of eosinophils in the normal range and there were no signs of eosinophilic infiltration in the lungs in the histopathology on the day of sacrifice, indicative of no association with vaccine- enhanced disease. IL-8, a key chemokine, responsible for the recruitment of neutrophils and other immune cells, was substantially high in vaccinated group III indicative of vaccine-induced immune response to infection.

Organ viral load, interstitial pneumonia and detection of viral antigen by IHC in lung tissue strengthens the evidence of SARS-CoV-2-induced pulmonary disease in placebo group animals. Group III (3 µg, adjuvant-A) animals had no clinical/radiological evidence of disease demonstrating significant protection from SARS-CoV-2. These findings correlate well with protection from disease as demonstrated by serological, gross pathological and HPE findings.

This study was performed during the period of countrywide lockdown in India with a complete suspension of international flights because of the pandemic that became detrimental to the procurement of reagents. This fact was a major limiting factor because of which detailed T-cell immunogenicity during vaccination and post-viral challenge period could not be performed. However, in spite of this limitation, the immunogenicity and protective efficacy have been amply demonstrated by the serological response (IgG, NAb), viral clearance from BAL fluid and respiratory tract tissues/organs and necropsy findings in the vaccinated animals compared to the placebo group.

Altogether, this study demonstrates that a two-dose vaccination regimen using 3-µg dose of the vaccine candidate with adjuvant B induces a significant immune response and provides effective protection in animals challenged with SARS-CoV-2.

## Methods

### Ethics statement

The study was approved by the Institutional Project Review Committee, and Institutional Biosafety Committee, ICMR-National Institute of Virology (NIV), Pune. The study was recommended by the Institutional Animal Ethics Committee (registration 43/GO/ReBi/SL/99/CPCSEA) and further approved by Committee for the Purpose of Control and Supervision of Experiments on Animals (CPCSEA), New Delhi letter No. V11011 (13)/7/2020-CPCSEA-DADF dated 08.06.2020. The permission from the Office of Principal Chief Conservator of Forests (PCCF), Maharashtra state, was obtained for capturing of 30 rhesus macaques from the wild. A decline in the macaque population was observed in the permitted areas of Maharashtra state, India, due to the habitat change of macaques probably imposed by food scarcity during the COVID-19 lockdown period of India (March 25, 2020 till May 31, 2020). Hence, twenty rhesus macaques (12 male and 8 female) were captured using experienced monkey catchers with the prior approval from PCCF, Maharashtra state, India. The approximate age of the animals was determined based on the dentition and body measurements. The research was conducted in compliance with the guidelines laid down by CPCSEA, Government of India^23^.

### Generation of vaccine

Vero CCL-81 cells were initially grown in tissue culture flasks and cell stacks using Dulbecco’s Modified Eagle Medium (DMEM) (Sigma-Aldrich, India) containing 10% newborn calf serum (NBCS) (Sigma-Aldrich, India). SARS-CoV-2 strain, NIV-2020-770, (GISAID ID: EPI_ISL_420545) isolated in Vero CCL-81 cells was used for the vaccine development^24^. The virus strain belongs to the G clade according to the GISAID classification and is 99.97% identical to the Wuhan Hu-1 strain^25^. G clade sequences are predominantly circulating in India and contribute to ~74% of the SARS-CoV-2 sequences circulating in the world^26–28^. The genetic stability of the NIV-2020-770 strain was demonstrated by multiple passages in Vero CCL-81 cells and by next-generation sequencing^7^. Virus propagation was done in a bioreactor (Corning Life Science) at the temperature of 36 ± 1 °C and was harvested at 36–72 h post infection and supernatants were collected, clarified and aliquoted. All the infectious work was performed in a biosafety level-3 laboratory. The virus was inactivated with ß-propiolactone at a ratio of 1:2500 at 2–8 °C for 24–32 h. It was further purified by column chromatography and concentrated using a tangential flow filtration system.

Three formulations of inactivated SARS CoV-2 vaccine candidates were prepared with two adjuvants (A and B): Algel 1 (aluminium hydroxide gel) and Algel 2 (imidazoquinoline, aTLR7/TLR8 agonist adsorbed on aluminium hydroxide gel). In this study, we have defined Algel 1 as Adjuvant A and Algel 2 as adjuvant B. Preliminary immunogenicity and safety study of BBV152 with Algel 1 and Algel 2, in various doses 3, 6 and 9 µg doses, were performed in mice, rat and rabbit model^7^. Based on the observations of this study, three candidate formulations (6 µg + Algel 1 (BBV152A), 3 µg + Algel 2 (BBV152B) and 6 µg + Algel 2 (BBV152C)) were chosen. Algel 2 was found eliciting high NAb titres and robust T-cell immunity. Further, these three candidate formulations were studied for their protective efficacy against SARS-CoV-2 in Syrian hamster model and all the candidates were found immunogenic and protective^8^.

### Study design and experiments on rhesus macaques

Rhesus macaques (*Macaca mulatta*) were housed in individual cages at the animal facility of ICMR-NIV, Pune. The animals were maintained on commercial pelleted feed, fruits, vegetables and ad libitum potable drinking water with a 12-h/12-h dark/light cycle. All the animals were clinically evaluated for skin/systemic disorders, haemoglobin, total leukocyte count, differential leukocyte count, platelet count, packed cell volume, biochemical parameters (AST, ALT, bilirubin, serum proteins, alkaline phosphatase, LDH, BUN, creatinine, cholesterol, triglycerides, sodium, potassium and glucose), abdominal ultrasonography, chest X-ray and tuberculin test and were found fit for the study. Animals were screened for Kyasanur forest disease virus and SARS-CoV-2 and IgG antibodies were found to be negative^29,30^. Biomedic data systems temperature transponder was implanted in the interscapular region subcutaneously for monitoring of body temperature during the study.

Twenty adult animals aged 3–12 years were divided into four groups of five animals (3 M, 2 F) each viz. placebo group, groups II, III and IV. The placebo group was administered phosphate buffer saline (PBS), groups II, III and IV were immunised with formulations of purified inactivated SARS-CoV-2 vaccine candidates: 6 μg + adjuvant-A, 3 μg + adjuvant-band 6 μg + adjuvant-B, respectively. Animals were administered with two doses of vaccine/placebo on days 0 and 14, respectively, intramuscularly in the deltoid region. Blood samples were collected on 0, 12, 19, 26 and 28 days for assessing anti-SARS IgG antibody and NAb titres (Fig. 1a).

After completion of 28 days of immunisation, animals  were shifted to animal biosafety level-4 facility. Animals were challenged with 1 ml of SARS-CoV-2 (P-3, NIV-2020770, TCID50 10^6.5^/ml)^24^ intratracheally and 0.25 ml in each nostril. Animals were monitored twice daily and clinical scoring was performed based on parameters as listed in Supplementary Table 1. Clinical examination was done on 0, 1, 3, 5 and 7 DPI along with body temperature, body weight, pulse rate and oxygen saturation at room air (Supplementary Table 1). NS, TS, rectal swab (RS), chest X-ray, blood specimens and BAL fluid were collected on 0, 1, 3, 5 and 7 DPI. BAL fluid collection was performed using a flexible paediatric bronchoscope (3.9 mm) (Pentax Medical India Private Limited) under general anaesthesia. The bronchoscope was inserted into the trachea and was guided through bronchus past the third bifurcation; 3–5 ml of saline was instilled at room temperature and aspiration was stopped after we had recovered 30–50% of the instilled volume. The weight of the monkeys in our study varied from 2.5 to 9.5 kilograms and hence installation of larger volumes of saline would have been detrimental to the health of the animals as bronchoscopy was being conducted every alternate day till 7 DPI. On 7 DPI during bronchoscopy prior to necropsy, we instilled a total volume of 10–15 ml of saline for BAL fluid collection.

During necropsy, the following organs: brain, nasal mucosa, tonsil, nasopharynx, oropharynx, cervical lymph node, trachea, lungs, mediastinal lymph node, heart, spleen, liver, kidneys, urinary bladder, gastrointestinal tract, skin along with underlying deltoid muscle from the immunisation site and cerebrospinal fluid (CSF) were collected.

### Enzyme-linked immunosorbent assay for detection of anti-SARS-CoV-2 IgG antibody

Immunoplates (Maxisorp, Nun) were coated with 100 µl/well of SARS-CoV-2 antigen overnight at 4 °C in the carbonate buffer. Subsequently, wells were blocked with liquid plate sealer (CANDOR Bioscience GmbH, Germany) for 2 h at room temperature) 25–30 °C. One-hundred microlitres of diluted rhesus macaque serum sample 1:100 in 1% bovine serum albumin in 1× PBS containing 0.1% Tween (PBST) were added to duplicate wells and incubated at 37 °C for one hour. To each well, 100 µl of a 1:15000 dilution of goat anti-monkey IgG peroxidase-conjugated antibodies (Sigma, USA) were added and incubated at 37 °C for 1 h. The plates were washed five times with a wash buffer (1× PBST) post all incubations. One-hundred microlitres of (3,3’5,5’-Tetramethylbenzidine (TMB) substrate was added and incubated for 10 min. The reaction was stopped by 1N H_2_SO_4_ and absorbance was measured at 450 nm^30^. The sample was considered positive when the P/N ratio was more than 1.5 and optical density (OD) values with the SARS-CoV-2 antigen were above 0.2.

### S1-RBD protein-specific SARS-CoV-2 IgG capture ELISA

Anti-SARS-CoV-2 S1 Receptor Binding Domain (RBD) protein IgG in the animal serum was captured by the viral recombinant RBD of S1 subunit of the Spike protein (S1-RBD) coated onto the wells. Serum samples were diluted at the ratio of 1:25, 1:100, 1:400, 1:1600, 1:6400, 1:25,600 and 1:102,400 for titre determination. Fifty microlitres of the diluted samples of each animal at 0 and 7 DPI, positive and negative control, were added to the respective wells. ELISA plate was incubated at 37 °C for 1 h followed by washing with wash buffer five times. Fifty microlitres of ready-to-use anti-monkey IgG HRP was added to each well and was incubated at 37 °C for 30 min. After washing, 100 µl of liquid TMB substrate added and incubated at room temperature in the dark for 10 min. The reaction was stopped with 100 µl of stop solution after 10 min. The absorbance was measured at 450 nm. If the OD value of sample tested exceeds 0.2 and sample OD/negative control OD > 2.0, the sample was considered positive.

### SARS-CoV-2-recombinant N-protein IgG capture ELISA

Anti-SARS-CoV-2 N protein IgG antibody in the animal serum was detected using recombinant N protein-coated wells. For determination of titre, serum samples were diluted fourfold from 1:25 to 1:102,400. Fifty microlitres of the diluted samples of each animal at 0 and 7 DPI, along with positive and negative control, were added to the respective wells. The ELISA plate was incubated at 37 °C for 1 h. Fifty microlitres of ready-to-use anti-monkey IgG HRP was added and was incubated at 37 °C for 30 min. Plates were washed five times using wash buffer containing 1× PBST after each incubation step. One-hundred microlitres of liquid TMB substrate was added and plates were incubated at room temperature in the dark for 10 min. The reaction was stopped with 100 µl of stop solution. The absorbance was measured at 450 nm. If the OD value of the sample tested exceeds 0.2 and sample OD/negative control OD > 2.0, the sample was considered positive.

### Plaque reduction neutralisation test

A fourfold serial dilution of rhesus macaque’s serum samples was mixed with an equal amount of virus suspension containing 50–60 plaque-forming units (PFU) in 0.1 ml. After incubating the mixtures at 37 °C for 1 h, each virus-diluted serum sample (0.1 ml) was inoculated onto one well of a 24-well tissue culture plate containing a confluent monolayer of Vero CCL-81 cells. After incubating the plate at 37 °C for 60 min, an overlay medium consisting of 2% carboxymethyl cellulose (CMC) with 2% foetal calf serum (FCS) in 2× MEM was added to the cell monolayer, and the plate was further incubated at 37 °C in 5% CO_2_ for 5 days. Plates were stained with1% amido black for an hour. Antibody titre was defined as the highest serum dilution that resulted in >50 (PRNT50) reduction in the number of plaques^31^. The serum samples of each animal were also tested with heterologous virus strain Q-100 and Q-111. Both these strains belong to unclassified clade isolated from COVID-19 positive patient’s samples from Iran^24^.

### Detection of SARS-CoV-2 genomic and subgenomic viral RNA

The organs harvested during necropsy were uniformly weighed and homogenised (Tissue homogenizer, Qiagen, Germany) using 1 ml of sterile MEM (GIBCO, Thermo Fisher Scientific, USA). Two-hundred microlitres of each specimen (NS, TS, rectal swab, whole blood, BAL fluid, urine, CSF and tissue homogenates) were used for RNA extraction using MagMAX™ Viral/Pathogen Nucleic Acid Isolation Kit (Thermo Fisher Scientific, USA). SARS-CoV-2 real-time RT-PCR was performed using a 25-µl reaction containing 5 µl of RNA, 12.5 μL of 2× reaction buffer provided with the Superscript III one-step RT-PCR system with Platinum Taq Polymerase (Invitrogen, Darmstadt, Germany), 1 µl of reverse transcriptase/Taq mixture from the kit, 1.5 µl of Primer and probe mix (Supplementary Table 4). Thermal cycling was performed at 55 °C for 30 min for reverse transcription, followed by 95 °C for 3 min and then 45 cycles of 95 °C for 15 s, 58 °C for 30 s. A standard curve was plotted using serially diluted tenfold (10^1^–10^10^) in vitro-transcribed RNA for E gene (gRNA)^32,33^. E sgRNA was amplified using real-time RT-PCR from the above specimens using primers as shown in Supplementary Table 4. Thermal cycling was performed at the same cycling conditions as mentioned for E gene. A standard curve was plotted using serially diluted tenfold (10^1^–10^10^) in vitro-transcribed E sgRNA^34^.

### Lymphocyte subset and cytokine/chemokine profile

To analyse phenotype and proportion of T helper, T cytotoxic and B cells, anti-coagulated (0.1 ml) whole blood was surface-stained with appropriate fluorochrome-conjugated antibodies along with their corresponding isotype controls. Two sets of sample tubes were prepared, one for T cell (CD3-FITC, CD8-PE and CD4-APC) and another for B cell (CD45-PerCP, CD3-FITC and CD20-PE). After incubation for 30 min at 4 °C in the dark, 2 ml of RBC lysing buffer were added to each tube, vortexed and incubated at room temperature for 12 min. Two millilitres of washing solution was added to each tube. The sample tubes were centrifuged at 200×*g* for 5 min, and the supernatant was carefully aspirated out. The cell pellets were suspended in 500 µl of wash buffer and vortexed. The levels of IL-2, IL-5, IL-6, IL-8, IFN-γ and TNF-α in the serum samples were analysed using BD cytometric bead array flex sets, as per the manufacturer’s instructions^35^. Cytokine levels were measured on a BD FACSCaliburTM flow cytometry (BD Biosciences) using BD CellQuestTM Pro software V5.1. The data were analysed using flow cytometric analysis program (FCAP) ArrayTM software V3. The detection sensitivities of IL-2, IL-5, IL-6, IL-8, IFN-γ and TNF-α were 11.2, 1.1, 1.6, 1.2, 1.8 and 1.2 pg/ml, respectively.

### Virus isolation from clinical/necropsy specimens

One-hundred microlitres of each specimen was inoculated onto 24-well Vero CCL-81 cell monolayers maintained in MEM (Gibco, UK) and incubated for 1 h at 37 °C with rocking every 10 min. Subsequently, media was removed and cells were washed with 1× PBS. Media with 2% FBS was added to each well and was incubated in a CO_2_ incubator at 37 °C for 5 days. The culture plate was examined daily for CPE using an inverted microscope (Nikon, Eclipse Ti, Japan). The cell culture supernatant from the wells showing CPE was further confirmed by real-time RT-PCR^24^.

### Histopathological examination and immunohistochemistry

Histopathological evaluation of all the six lobes of lungs of animals was performed on 7 DPI after necropsy. Tissue sections from lungs were immersion-fixed in 10% neutral buffered formalin. The fixed tissues were processed using an automated tissue processor using alcohol–xylene protocol for dehydration and clearing. The processed tissues were embedded in paraffin and were sectioned to 4–5 µm-thick sections using an automated microtome (Leica, Germany). The cut tissue sections were stained by haematoxylin and eosin^36^. The factors considered and reporting criteria used were as per the recommendation of the Working Group of the Society of Toxicologic Pathology’s Scientific and Regulatory Policy Committee^37^. Seven parameters were assessed during the histopathological examination of slides for lung sections in all animals for assessing the severity of disease in the lungs as mentioned in Supplementary Table 2. The parameters listed in Supplementary Table 2 were graded as no abnormality detected (NAD), minimal (+1), mild (+2), moderate (+3) or severe (+4) for each lung. Subsequently, the extent of disease for each lobe of animals was opined upon as mild, moderate and severe. Further, the analysis was done on the severity of infection depending on the number of lobes having evidence of disease for animals of placebo and vaccinated groups. Anti-SARS-CoV-2 immunoreactivity in the lung tissues was assessed using mouse polyclonal serum. For IHC, mouse polyclonal serum was used as the primary antibody (1:500) and anti-mouse HRP antibody was used as secondary antibody^29,36^.

### Statistical analysis

Clinical, virological, haematological, biochemical and immunological data were analysed using GraphPad Prism software version 8.4.3 (GraphPad, San Diego, California) and Stata software version 14. (StataCorp LLC, USA). The clinical, virological and serological data for the different groups were initially compared using the nonparametric Kruskal–Wallis test. The groups that were significant using the Kruskal–Wallis test were further assessed using the Mann–Whitney test. A group-wise comparison was performed to assess significance between the placebo and the other vaccinated groups using a two-tailed Mann–Whitney test. The *P* values < 0.05 were considered significant and are marked on the figures. Nonsignificant values are not depicted in the figures. The log10 plots below the detection limits are depicted as 1 for the illustration purpose. The detection limits are marked with the dotted lines on the respective figures.

### Reporting summary

Nature research reporting summary linked to this paper contains the information on research design. Further information on research design is available in the Nature Research Reporting Summary linked to this article.

## Supplementary information

Supplementary Information

Reporting Summary

## Data Availability

Data are available in the paper and in the Supplementary Material. All other relevant data are available from the authors. Source data are provided with this paper.

## Source data

Source Data
