# Supplementary material for: Immunogenicity and protective efficacy of inactivated SARS-CoV-2 vaccine candidate, BBV152 in rhesus macaques
Source: Nat Commun. 2021 Mar 2;12:1386. doi: 10.1038/s41467-021-21639-w (PMC7925524; doi:10.1038/s41467-021-21639-w)
Supplement: Supplementary file 1 — Supplementary Information [file 41467_2021_21639_MOESM1_ESM.pdf]

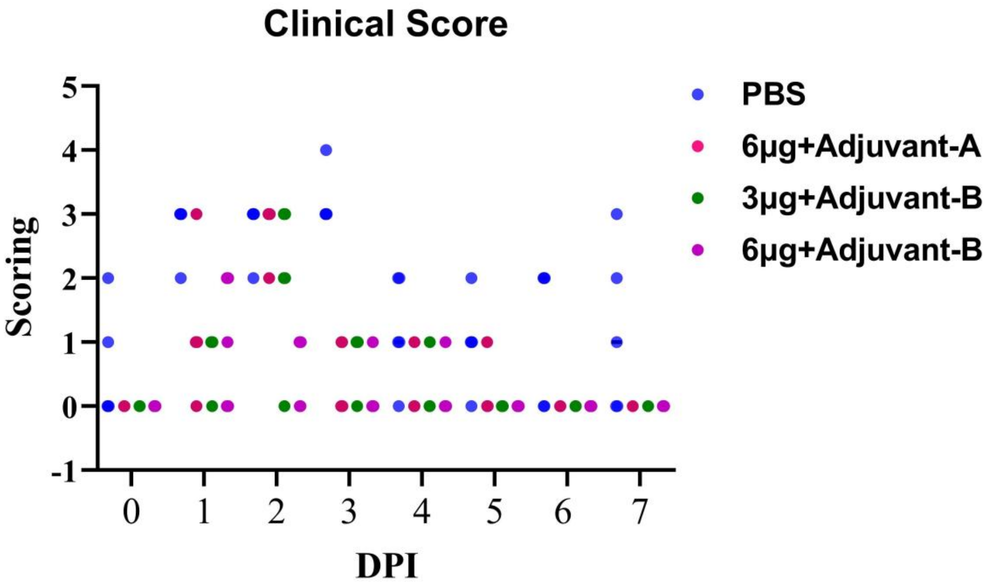

Supplementary figure. 1| Scatter plot for clinical scores of vaccinated macaques post-challenge. The animals of were scored as per the parameters described in Supplementary table 2 and observations mentioned in Supplementary table 3. Group I = blue, group II = pink, group III = green and group IV = purple, number of animals = 5 animals in each group. Source data are provided as a Source Data file.

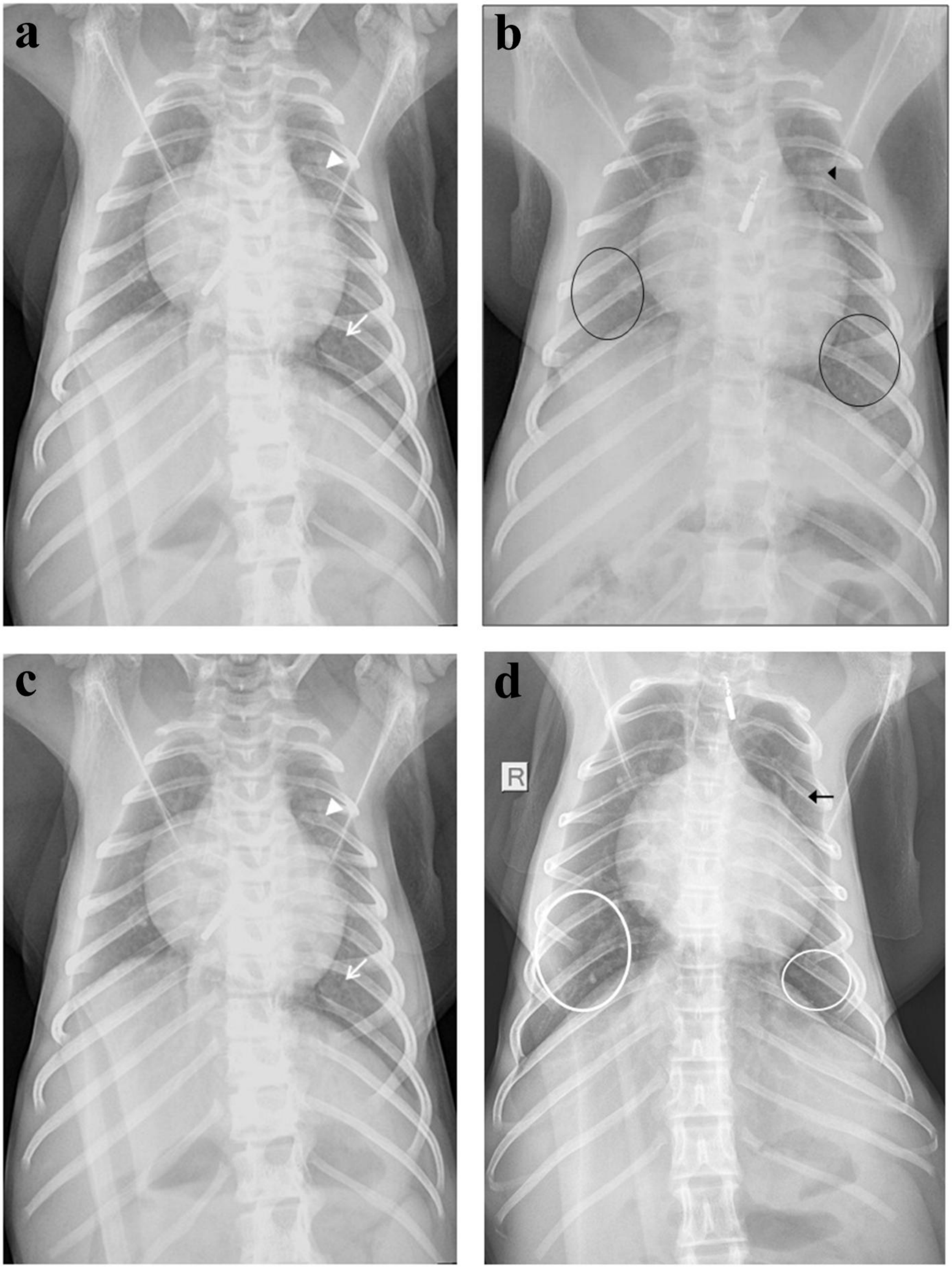

a
b
c
d
R

Supplementary figure. 2| Anteroposterior (AP) chest radiographs obtained from different animals. (a) Normal chest X-ray (Group III at 7 DPI). (b) Infiltrate in left upper lobe (black arrowhead), bronchopneumonia in lower lobes of both the lungs (black circles) (Group I – Placebo at 7 DPI). (c) Infiltrate in left upper lobe (white arrowhead) and lobar consolidation in left lower lobe (white arrow) (Group II at 3 DPI. The lesions resolved by 5 DPI) (d) Lobar consolidation in left upper lobe (black arrow) with bilateral lower lobe bronchopneumonia (white circles) (Group IV at 3 DPI. The lesions resolved by 5 DPI). 'R' represents the position indicator for the right side of the animal.

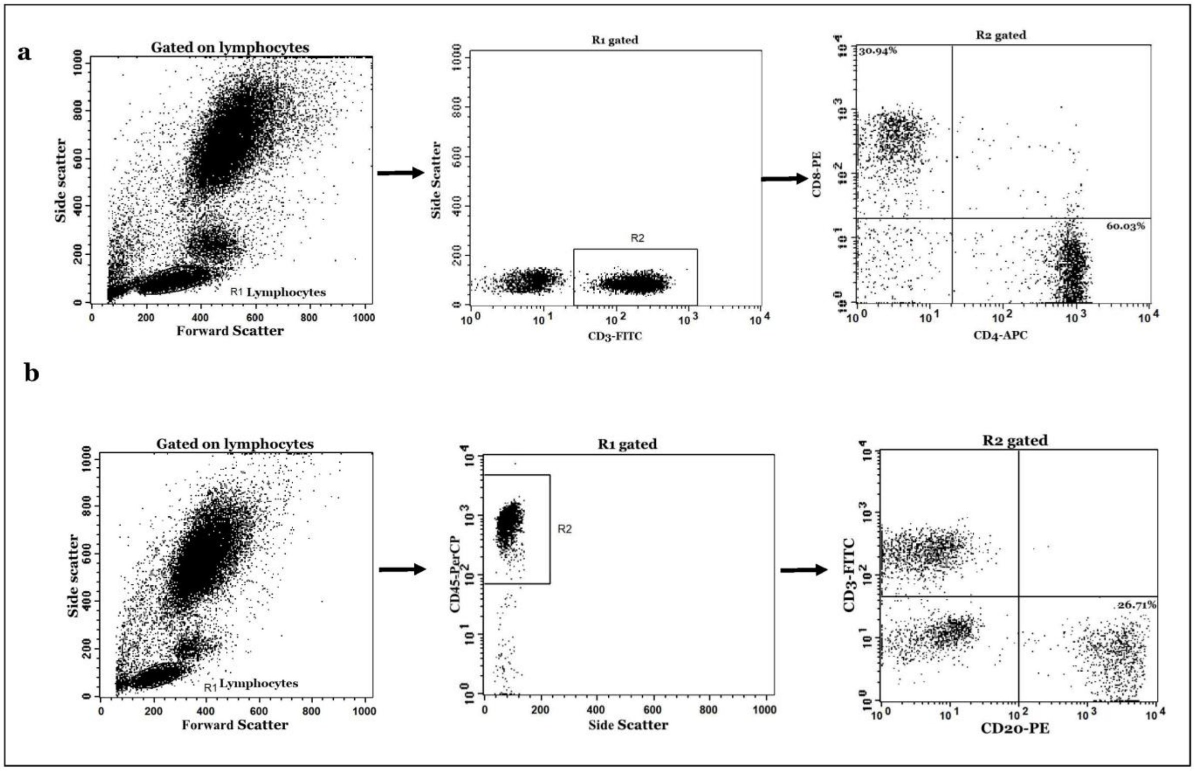

Supplementary figure. 3| T lymphocyte profile in animals of each study group on 0,1,3,5 and 7 days post-infection (DPI). Gating strategy to determine the percentages of lymphocytes and T cell subsets **(a)** and B cells **(b)** in the peripheral blood of rhesus monkey.

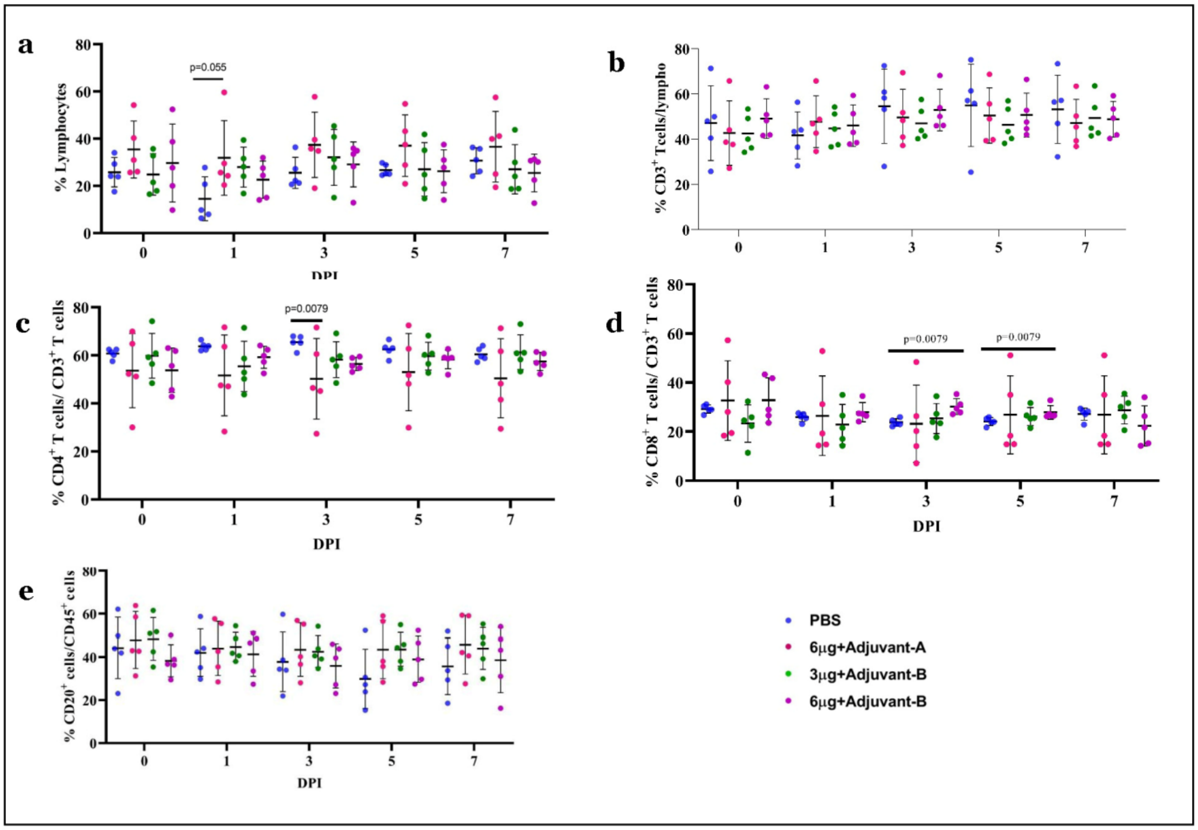

Supplementary figure. 4| T lymphocyte profile in animals of each study group on 0,1,3,5 and 7 DPI. Lymphocytes (a) were gated on the basis of forward- and side-scatter and CD3+ T cells (b) were gated on lymphocytes (R1). CD4 + cells (c) and CD8 + cells (d) were gated on CD3+ T cells (R2). Similarly, for B cells, lymphocytes were gated on the basis of forward- and side-scatter and CD45+ cells were gated on lymphocytes (R1). CD20 + B cells (e) were gated on CD45+ cells (R2). Horizontal lines indicate mean values. Data are presented as mean values +/- standard deviation (SD). Statistical comparison was done by comparing the vaccinated group with the placebo group as control. Group I = blue, group II = pink, group III = green and group IV = purple, number of animals = 5 animals in each group. Source data are provided as a Source Data file.

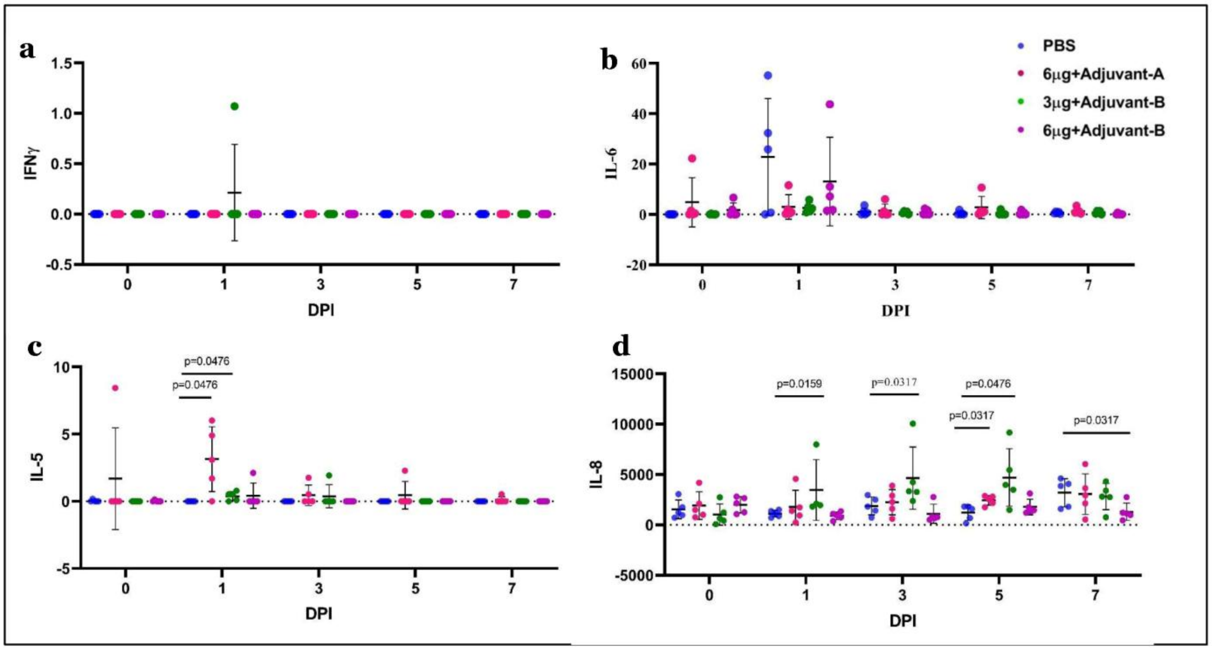

Supplementary figure. 5| Cytokine profile in animals of at 0,1,3,5 and 7 days post-infection (DPI). (a) IFN-γ (b) IL-6 (c) IL-5 (d) IL-8. Data are presented as mean values +/- standard deviation (SD). Statistical comparison was done by comparing the vaccinated group with the placebo group as control. Group I = blue, group II = pink, group III = green and group IV = purple, number of animals = 5 animals in each group. Source data are provided as a Source Data file.

Supplementary table 1: Clinical scoring sheet

Group	ID	0 DPI	1 DPI	2 DPI	3 DPI	4 DPI	5 DPI	6 DPI	7 DPI
**I**	1	Normal	Reduced food and water intake, lethargy	Reduced water intake, lethargy	Reduced food and water intake lethargy	Lethargy	Lethargy	Normal	Normal
	2	Normal	Reduced food and water intake, lethargy	Reduced self grooming, food and water intake, lethargy	Reduced self grooming food and water intake, lethargy	Normal	Normal	SpO2-86%, PR-140 /min	SpO2-95% PR-115/min
	3	Reduced water intake	Reduced food intake, lethargy	Reduced food and water intake, lethargy	Reduced food and water intake, lethargy	Reduced water intake	Reduced water intake	Normal	Normal
	4	Normal	Reduced food and water intake, lethargy	Ruffled hair coat, reduced food intake and vocalization	Ruffled hair coat, reduced food and water intake, lethargy	Ruffled hair coat, lethargy	Ruffled hair coat	SpO2-88% PR-160 /min	SpO2-93% PR-160/min
	5	Reduced water intake	Reduced food and water intake	Reduced food and water intake lethargy	Reduced food and water intake, lethargy	Reduced water intake, lethargy	Reduced water intake, lethargy	Reduced water intake, lethargy	Lethargy SpO2-89% PR-114 /min
**II**	6	Normal	Normal	Reduced self grooming, food and water intake	Reduced water intake	Normal	Normal	Normal	Normal
	7	Normal	Reduced water intake	Reduced self grooming, food and	Normal	Normal	Normal	Normal	Normal

				water intake					
	8	Normal	Reduced food intake	Reduced self grooming and food intake	Normal	Normal	Normal	Normal	Normal
	9	Normal	Reduced food intake	Reduced food and water intake, lethargy	Normal	Normal	Normal	Normal	Normal
	10	Normal	Reduced food intake and self grooming lethargy	Reduced self grooming, food and water intake	Reduced water intake	Reduced water intake	Reduced water intake	Normal	Normal
**III**	11	Normal	Reduced water intake	Lethargy, reduced food intake	Reduced water intake	Normal	Normal	Normal	Normal
	12	Normal	Normal	Normal	Normal	Normal	Normal	Normal	Normal
	13	Normal	Normal	Reduced food and water intake	Reduced water intake	Normal	Normal	Normal	Normal
	14	Normal	Reduced water intake	Lethargy, reduced food and water intake	Reduced water intake	Normal	Normal	Normal	Normal
	15	Normal	Reduced water intake	Lethargy, reduced food and water intake	Reduced water intake	Reduced water intake	Normal	Normal	Normal
**IV**	16	Normal	Normal	Normal	Normal	Normal	Normal	Normal	Normal
	17	Normal	Lethargy, Reduced water intake	Lethargy	Normal	Normal	Normal	Normal	Normal
	18	Normal	Normal	Normal	Normal	Normal	Normal	Normal	Normal
	19	Normal	Lethargy, reduced water intake	Lethargy	Normal	Normal	Normal	Normal	Normal
	20	Normal	Reduced water intake	Reduced water intake	Reduced water intake	Reduced water intake	Normal	Normal	Normal

*****DPI-Days post-infection, SpO2- Peripheral capillary oxygen saturation at room air, PR-Pulse rate, mins-minutes

Supplementary table 2: Parameters considered during histopathological evaluation. The mean of number of lobes out of the six lobes evaluated was calculated for each group with the range given in brackets.

Sr. No.	Parameter	Group I	Group II	Group III	Group IV
		**Mean affected number of lung lobes (Range of no. of lung lobes affected)**			
1.	Vascular changes characterized by congestion and hemorrhages in the lung parenchyma	3.4 (0-6)	1.0 (0-2)	0.8 (0-1)	2.0 (0-5)
2.	Bronchial pathological changes characterized by the degeneration and necrosis with loss of bronchial epithelium	3.4 (1-5)	1.2 (0-2)	1.0 (0-3)	1.2 (0-4)
3	Hyaline change characterized by the presence of exudate at alveolar septa (epithelial wall) and lumen of alveoli.	2.8 (1-6)	0.6 (0-1)	0.4 (0-1)	1.0 (0-2)
4	Alveolar pathological changes characterized by consolidation and thickening of alveolar septa with Type II-Pneumocyte hyperplasia	3.2 (0-6)	1.4 (0-2)	1.2 (0-4)	1.8 (0-3)
5	Edematous changes and fibrin deposition in the lung parenchyma/alveolar lining	2.8 (0-6)	0.6 (0-1)	0.6 (0-1)	0.4 (0-1)
6	Thickening of alveolar septa characterized by the infiltration of inflammatory cells in the interstitial space	4.4 (1-6)	2.8 (0-4)	0.8.(0-1)	1.2 (0-4)
7	Perivascular and peribronchiolar cuffing characterized by infiltration of mononuclear/inflammatory cells in peribronchial and perivascular areas of lung	2.8 (0-5)	0.6 (0-1)	0.2 (0-1)	0.4 (0-1)

Supplementary table 3: Final grade of involvement of each lobe of lung with cumulative score for each animal of vaccinated and placebo group{No abnormality detected (NAD), minimal changes (1+), mild changes (2+), moderate changes (3+) and severe changes (4+)}

Group	Animal ID	Right upper lobe	Right middle lobe	Right lower lobe	Left upper lobe	Left middle lobe	Left lower lobe	Histopathological scores
**Group I**	**1**	Minimal (+1)	Mild (+2)	Minimal (+1)	Minimal (+1)	Mild (+2)	Mild (+2)	**9**
	**2**	NAD	Mild (+2)	NAD	Minimal (+1)	NAD	Minimal (+1)	**4**
	**3**	Minimal (+1)	Minimal (+1)	Mild (+2)	Mild (+2)	NAD	Moderate (+3)	**9**
	**4**	Minimal (+1)	NAD	Mild (+2)	Minimal (+1)	NAD	Minimal (+1)	**6**
	**5**	NAD	NAD	Minimal (+1)	Mild (+2)	Minimal (+1)	Mild (+2)	**6**
**Group II**	**6**	NAD	NAD	NAD	NAD	NAD	Minimal (+1)	**1**
	**7**	NAD	NAD)	Mild (+2)	NAD	NAD	NAD	**2**
	**8**	NAD	NAD	Minimal (+1)	NAD	NAD	NAD	**1**
	**9**	Minimal (1+)	NAD	NAD	NAD	Minimal (+1)	NAD	**2**
	**10**	NAD	Minimal (+1)	NAD	NAD	NAD	NAD	**1**
**Group III**	**11**	NAD	NAD	NAD	Minimal (+1)	NAD	Minimal (+1)	**2**
	**12**	NAD	NAD	NAD	NAD	NAD	NAD	**0**
	**13**	NAD	Minimal (+1)	NAD	NAD	NAD	NAD)	**1**
	**14**	NAD	Minimal (+1)	NAD	NAD	NAD	NAD	**1**
	**15**	NAD	NAD	NAD	NAD	NAD	Minimal (+1)	**1**
**Group IV**	**16**	Minimal (+1)	NAD	NAD	NAD	NAD	Minimal (+1)	**2**
	**17**	NAD	NAD	Minimal (+1)	NAD	NAD	NAD	**1**
	**18**	NAD	Minimal (+1)	NAD	NAD	Minimal (+1)	Minimal (+1)	**3**
	**19**	NAD	NAD	Minimal (+1)	NAD	NAD	Minimal (+1)	**2**
	**20**	NAD	Minimal (+1)	NAD	NAD	NAD	NAD	**1**

(* NAD= No Abnormality Detected)

Supplementary table 4: Details of Primers and probes used in Real time RT-PCR

Name	Oligo Type	Target gene	5'-Fluorophore	Sequences	3' Quencher	References
E_Sarbaco_F1	Forward Primer	Envelope	-	ACAGGTACGTTAATAGTTAATAGCGT	-	[33]
E_Sarbaco_R2	Reverse Primer	Envelope	-	ATATTGCAGCAGTACGCACACA	-	[33]
E_Sarbaco_P1	Hydrolysis probe	Envelope	FAM	ACACTAGCCATCCTTACTGCGCTTCG	BHQ-1	[33]
Sub.CoV_2_ E_For	Forward Primer	Envelope	-	CGATCTCTTGTAGATCTGTTCTCT	-	[35]
Sub.CoV_2_ E_Rev	Reverse Primer	Envelope	-	ATATTGCAGCAGTACGCACACA	-	[35]
Sub.CoV_2_ E_Probe	Hydrolysis probe	Envelope	FAM	ACACTAGCCATCCTTACTGCGCTTCG	BHQ-1	[35]

*FAM- Fluorescein, BHQ- Black hole quencher
