# Supplementary material for: Immunogenicity and protective efficacy of inactivated SARS-CoV-2 vaccine candidate, BBV152 in rhesus macaques
Source: Nat Commun. 2021 Mar 2;12:1386. doi: 10.1038/s41467-021-21639-w (PMC7925524; doi:10.1038/s41467-021-21639-w)
Supplement: Supplementary file 2 — Reporting Summary [file 41467_2021_21639_MOESM2_ESM.pdf]

nature research

Corresponding author(s): Dr. Balram Bhargava
Last updated by author(s): Jan 25, 2021

Reporting Summary

Nature Research wishes to improve the reproducibility of the work that we publish. This form provides structure for consistency and transparency in reporting. For further information on Nature Research policies, see our Editorial Policies and the Editorial Policy Checklist.

Statistics

For all statistical analyses, confirm that the following items are present in the figure legend, table legend, main text, or Methods section.

n/a	Confirmed	
☐	☒	The exact sample size (n) for each experimental group/condition, given as a discrete number and unit of measurement
☐	☒	A statement on whether measurements were taken from distinct samples or whether the same sample was measured repeatedly
☐	☒	The statistical test(s) used AND whether they are one- or two-sided<br>*Only common tests should be described solely by name; describe more complex techniques in the Methods section.*
☒	☐	A description of all covariates tested
☐	☒	A description of any assumptions or corrections, such as tests of normality and adjustment for multiple comparisons
☐	☒	A full description of the statistical parameters including central tendency (e.g. means) or other basic estimates (e.g. regression coefficient) AND variation (e.g. standard deviation) or associated estimates of uncertainty (e.g. confidence intervals)
☐	☒	For null hypothesis testing, the test statistic (e.g. F, t, r) with confidence intervals, effect sizes, degrees of freedom and P value noted<br>*Give P values as exact values whenever suitable.*
☒	☐	For Bayesian analysis, information on the choice of priors and Markov chain Monte Carlo settings
☒	☐	For hierarchical and complex designs, identification of the appropriate level for tests and full reporting of outcomes
☒	☐	Estimates of effect sizes (e.g. Cohen's d, Pearson's r), indicating how they were calculated

Our web collection on statistics for biologists contains articles on many of the points above.

Software and code

Policy information about availability of computer code

Data collection	Data collection for flow cytometric analysis was carried out using ArrayTM software V3.
Data analysis	Data analysis was done using the Graphpad Prism V8.3.0, Stata software V14 and BD CellQuestTM Pro software V5.1.

For manuscripts utilizing custom algorithms or software that are central to the research but not yet described in published literature, software must be made available to editors and reviewers. We strongly encourage code deposition in a community repository (e.g. GitHub). See the Nature Research guidelines for submitting code & software for further information.

Data

Policy information about availability of data

All manuscripts must include a data availability statement. This statement should provide the following information, where applicable:

- Accession codes, unique identifiers, or web links for publicly available datasets
- A list of figures that have associated raw data
- A description of any restrictions on data availability

All data are available in the manuscript or the supplementary material. Correspondence and requests for materials should be addressed to Dr. Balram Bhargava (balrambhargava@yahoo.com).

Field-specific reporting

Please select the one below that is the best fit for your research. If you are not sure, read the appropriate sections before making your selection.

☒ Life sciences ☐ Behavioural & social sciences ☐ Ecological, evolutionary & environmental sciences

For a reference copy of the document with all sections, see nature.com/documents/nr-reporting-summary-flat.pdf

Life sciences study design

All studies must disclose on these points even when the disclosure is negative.

Sample size

The sample size was determined based on the published data (Chandrashekhar et al. Science 2020) with nonhuman primate models for SARS-CoV-2, where the numbers used were sufficient for statistical analyses. Chandrashekar et al. and Deng et al. initially generated rhesus macaque models of SARS-CoV-2 infection and tested whether natural SARS-CoV-2 infection could result in immunity to viral re-challenge. [Chandrashekar, A. et al. SARS-CoV-2 infection protects against rechallenge in rhesus macaques. Science 80, 4776]

Data exclusions

No data were excluded from the analysis.

Replication

Histopathology and histochemistry: Multiple tissue sections were evaluated of each animal on necropsy day; [n=15 (vaccinated) or 5 (control).
Lymphocyte subset and cytokine analysis: Whole blood and serum samples were analyzed in duplicate from each animal for each time-point; n=15 (vaccinated) or 5 (control).
Serological analysis: Serum samples were analyzed in duplicate from each animal for each time-point; n=15 (vaccinated) or 5 (control).
qRT-PCR analysis: Nasal swab, throat swab, rectal swab, bronchoalveolar lavage fluid, urine, stool and tissue specimens were tested from each animal for each time-point; n=15 (vaccinated) or 5 (control).
All the attempts at replication were successful.

Randomization

All the rhesus macaques were randomly assigned to experimental groups. Randomization of the samples was done by assigning a random number to all samples and further sorting them according to the random numbers.

Blinding

Blinding was done for the following personnel:
- The investigators and co-investigators to group allocation during data collection of the laboratory experiments
- Person observing the animals daily
- Veterinary pathologists reviewing histopathology
- Clinical veterinarians performing exams
- Laboratory personnels performing the laboratory analysis

Reporting for specific materials, systems and methods

We require information from authors about some types of materials, experimental systems and methods used in many studies. Here, indicate whether each material, system or method listed is relevant to your study. If you are not sure if a list item applies to your research, read the appropriate section before selecting a response.

Materials & experimental systems

n/a	Involved in the study	
☐	☒	Antibodies
☐	☒	Eukaryotic cell lines
☒	☐	Palaeontology and archaeology
☐	☒	Animals and other organisms
☒	☐	Human research participants
☒	☐	Clinical data
☒	☐	Dual use research of concern

Methods

n/a	Involved in the study	
☒	☐	ChIP-seq
☐	☒	Flow cytometry
☒	☐	MRI-based neuroimaging

Antibodies

Antibodies used

Rabbit polyclonal anti-Monkey IgG , peroxidase-labeled, dilution 1:10000 (Sigma) cat # A2054 Lot No - 017M4771V
CD3-FITC, BD, cat # 556611, clone SP34
CD4-APC, BD, cat # 551980, clone L200
CD8-PE, BD, cat # 557086, clone RPA-T8
CD45-PerCP, BD, cat # 558411, clone D058-1283
CD20-PE, BD, cat # 555623, clone 2H7
IL-2 CBA flex set, BD, cat # 558270
IL-5 CBA flex set, BD, cat # 558278
IL-8 CBA flex set, BD, cat # 558277

	IL-IFN-γ CBA flex set, BD, cat # 558269<br>IL-6 CBA flex set, BD, cat # 558276<br>TNF-α CBA flex set, BD, cat # 560112
Validation	Rabbit polyclonal anti-Monkey IgG (whole molecule)–Peroxidase antibody used to detect and quantitate the level of IgG in monkey serum and biological fluids via chromogenic, chemoluminescent or fluorogenic immunochemical or immunohistochemical techniques These antibodies are indigenously validated against SARS-CoV-2 at ICMR-NIV Pune.

Eukaryotic cell lines

Policy information about cell lines

Cell line source(s)	Vero CCL-81 cells, ICMR-National Institute of Virology, Pune, India (not commercial)
Authentication	Cells are not authenticated in-house.
Mycoplasma contamination	Repeated testing was done to check the Mycoplasma contamination and found negative.
Commonly misidentified lines<br>(See ICLAC register)	Misidentified cell lines were not used in this study.

Animals and other organisms

Policy information about studies involving animals; ARRIVE guidelines recommended for reporting animal research

Laboratory animals	Rhesus macaques (Macaca mulatta), adult (3-12 years), 12 males, 8 female
Wild animals	Twenty wild-rhesus macaques [adult (3-12 years), 12 males, 8 female] were captured using experienced monkey catchers with the prior approval from the Office of Principal Chief Conservator of Forests (PCCF), Maharashtra state. All the possible point of contact have been appropriately taken care to mitigate the risk before and during transport to ICMR-NIV, Pune. Further, they were housed in the individual cages at the animal house, ICMR-NIV, Pune till the completion of immunization and were shifted to animal biosafety level-4 of Maximum Containment Facility before the challenge with SARS-CoV-2.<br>Macaques were euthanized, and lung, brain, nasal mucosa, tonsil, nasopharynx, oropharynx, cervical lymph node, trachea, lungs, mediastinal lymph node, heart, spleen, liver, kidneys, urinary bladder, gastrointestinal tract and skin along with underlying deltoid muscle from the immunization site and cerebrospinal fluid (CSF) were collected at 7 day post-infection. All the tissue specimens were screened for viral genomic and subgenomic RNA using qRT-PCR. Histopathology and immunohistochemistry analysis was performed on multiple lobes of the lungs.
Field-collected samples	The wild-caught rhesus macaques [adult (3-12 years), 12 males, 8 female] were housed in the individual cages at the animal house, ICMR-NIV, Pune till the completion of immunization and were shifted to animal biosafety level-4 of Maximum Containment Facility before the challenge with SARS-CoV-2. The rooms were maintained with appropriate temperature (20-27degree C), humidity (40-70%), ventilation (10-15 air changes/hour), light (150-300 lux), odor (<20ppm of ammonia) and noise levels (<60 dB). The animals were maintained on commercial pelleted feed, fruits, vegetables, and ad-libitum potable drinking water with a photo-period of 12hr/12hr dark/light cycle. The animals were monitored twice daily by qualified and trained personnel. All the animals were sacrificed at the end of the experiment.
Ethics oversight	The present study was approved by the Institutional project review committee, and Institutional Biosafety Committee, ICMR-National Institute of Virology (NIV), Pune. It was also recommended by the Institutional Animal Ethics Committee (Registration 43/GO/ReBi/SL/99/CPCSEA)of ICMR-NIV, Pune which was further approved by Committee for the Purpose of Control and Supervision of Experiments on Animals (CPCSEA), New Delhi letter No. V11011 (13)/7/2020-CPCSEA-DADF dated 08.06.2020. The permission from Office of the Principal Chief Conservator of Forests (PCCF), Maharashtra state, was also obtained for the trapping of the required number of rhesus macaques from the wild. The research was conducted in compliance with the guidelines laid down by CPCSEA, Government of India.

Note that full information on the approval of the study protocol must also be provided in the manuscript.

Flow Cytometry

Plots

Confirm that:

☒ The axis labels state the marker and fluorochrome used (e.g. CD4-FITC).

☒ The axis scales are clearly visible. Include numbers along axes only for bottom left plot of group (a 'group' is an analysis of identical markers).

☒ All plots are contour plots with outliers or pseudocolor plots.

☒ A numerical value for number of cells or percentage (with statistics) is provided.

Methodology

Sample preparation	Cell phenotyping: A well-mixed anti-coagulated whole blood was surface-stained with appropriate fluorochrome-conjugated antibodies along with their isotype controls. After 30 min incubation for at 4 degree celsius, 2 ml of RBC lysing buffer (BD, cat # 349202) were added to each tube, incubated at RT for 12 minutes, washed and the cell pellets were suspended in 500µl wash buffer.<br><br>Cytokine analysis: A 50µl serum sample was incubated with capture beads coupled with antibodies. The immune complexes were incubated with detection antibody conjugated with phycoerythrin. The standards provided by the manufacturer were used to prepare the standard curve for each cytokine. The level of cytokines in the serum samples were analyzed using BD Cytometric bead array flex sets, as per manufacturer's instructions.
Instrument	BD FACSCaliburTM
Software	BD CellQuestTM Pro software V 5.1
Cell population abundance	An acquisition threshold was set at a minimum of 50000 events in the lymphocyte gate
Gating strategy	Lymphocyte populations were gated based on FSC-H and SSC-H properties. From this lymphocyte, CD3+ T-cells were gated to determine percentage of CD4+ and CD8+ T-cells. Similarly, CD45+ cells were gated on lymphocytes and percentage of CD20+ B cells were determined.

☒ Tick this box to confirm that a figure exemplifying the gating strategy is provided in the Supplementary Information.
